# A qualitative study on reasons for early removal of Implanon among users in Arba Minch town, Gamo Goffa zone, South Ethiopia: a phenomenological approach

**DOI:** 10.1186/s12905-019-0876-1

**Published:** 2020-01-02

**Authors:** Mesfin Mamo Utaile, Mesfin Kote Debere, Etsehiwot Tilahun Nida, Dube Jara Boneya, Amsale Tekle Ergano

**Affiliations:** 1 Department of Public Health, College of Medicine and Health Sciences, Arba Minch University, Arba Minch, Ethiopia; 2 School of Public Health, College of Health Sciences, Addis Ababa University, Addis Ababa, Ethiopia; 3 Department of Public Health, College of Medicine and Health Sciences, Debre Markos University, Debre Markos, Ethiopia; 4 Department of Economics, College of Business and Economics, Arba Minch University, Arba Minch, Ethiopia

**Keywords:** Early removal of Implanon, Qualitative study, Phenomenological approach, South Ethiopia

## Abstract

**Background:**

Implanon is one of the cost - effective long acting reversible contraceptive methods used for spacing and limiting births in Ethiopia. Despite the scaling up initiative undertaken by the Ethiopian Government, Implanon uptake is very low compared to short acting contraceptive methods. There is low utilization of Implanon with high level of discontinuation in Ethiopia. Therefore, this study was conducted to explore the reasons for early removal of Implanon among users in Arba Minch town, South Ethiopia.

**Methods:**

A community-based qualitative exploratory study using phenomenological approach was conducted. In-depth and key informant interviews were used to collect data from April 20–27, 2018 in Arba Minch town. Convenient sampling was employed to recruit participants from the households of targeted villages. A total of 10 in-depth interviews with women who recently removed Implanon and 5 key informant interviews with health extension workers were conducted. The sample size was determined based on the concept of saturation. The collected data were analyzed using thematic content analysis technique. Data coding and analysis were facilitated by using Open code version 4.0 software.

**Results:**

This study revealed that majority of participants were able to mention at least three types of contraceptive methods available in the nearby health facilities. The study underlined that side effect of the method, husband opposition, seeking more children, and method failure were the common reasons for early removal of Implanon, in which side effect of the method was the main reason. Among various forms of side effects of Implanon identified by users, heavy and irregular bleeding was mentioned as the most frequently occurring side effect.

**Conclusion:**

Our result indicated that heavy and irregular bleeding was the main reason for early removal of Implanon. Therefore it suggests improvement in the service delivery system. Improving client’s education and counseling service program could contribute much to avoid unreasonable and untimely removal of Implanon.

## Background

Obstetric complications are the leading causes of disability and death among women of reproductive age in poor resource countries [1]. Almost all of the maternal deaths (99%) occur in developing countries with sub-Saharan Africa (SSA) alone accounting for approximately 66% [2, 3]. Though maternal mortality is declining in Ethiopia, the progress is at a slow rate [4–7]. The Ethiopian Demographic and Health Survey (EDHS) report of 2016 indicated that Maternal Mortality Ratio (MMR) was estimated to be 412 per 100,000 live births [7]. By considering the adverse health consequences of obstetric complications, the Ministry of Health (MOH) has applied a multi-pronged approach to reduce maternal morbidity and mortality [8].

In Ethiopia, in spite of various programs and services that are in place to improve maternal health, the records still signifies it as serious public health concern [6, 9, 10]. Furthermore, the causes and factors of maternal health problems are diverse and multifaceted [9–12]. These problems are in part due to low utilization of maternal health care services including family planning [6].

Family planning (FP) is a low cost yet effective method of preventing maternal health problems in reducing high risk births. It is defined as the ability of couples to space and limit their births. Usually, it is realized through contraceptive uses and correction of involuntary infertility [12–14]. Contraceptive methods are categorized into two groups. These are long - acting and permanent methods and short - acting methods. Long - acting methods can be used for both limiting and spacing purposes, while permanent methods can be used for limiting only. Short-acting methods are more appropriate for women who want to space childbearing [15].

According to EDHS report of 2016, the total fertility rate (TFR) in Ethiopia has declined to 4.6 children per woman. Nevertheless, the difference among rural and urban women is remarkable. On average, rural women have nearly three more children than urban women [7]. The overall decline in fertility might be attributed to the diversity and expansion of FP services made by the health sector [16].

Currently in Ethiopia, the contraceptive prevalence rate is 36%. Among married women, 35% are using modern contraceptive methods, in which injectables (23%), implants (8%), IUD (2%) and the pill (2%) were popular methods. Despite the effectiveness and safety of long - acting reversible contraceptives, the actual uptake in resource – poor settings including Ethiopia is low compared to short - acting methods [7, 17–19].

Implanon, the log acting reversible sub-dermal contraceptive implant, is a single rod that contains 68 mg Etonogestrel to offer contraception for 3 years. It is an implant which does not depend on user adherence for effectiveness [20, 21]. In Ethiopia, in 2009, the FMOH made a strategic decision to launch an implant scale-up initiative. To achieve this goal, the FMOH decided to make Implanon available at the community level as it is easy to administer. Despite the effort put by FMOH to scale up Implanon, only 8% of currently married women are using it [7]. Besides its low utilization, the discontinuation rate is between 16 and 46.5% in some African countries including Ethiopia [22–24].

Implanon discontinuation is defined as discontinuation at less than 2.5 years after insertion of implanon [23]. Different literature reported that the major reasons for Implanon discontinuation are side effects, health concerns, desire to become pregnant and partner opposition. Among side effects, menstrual disturbance is the most common reason [23–25].

Studies in the Northern part of Ethiopia revealed that shifting to other methods, inconvenience to use, religious and contraceptive failure were the main reasons for Implanon removal [23, 24]. Partner influence, discomfort on the insertion area and cultural influences are also among the major reasons for early removal [25]. Several studies have identified factors affecting use of FP methods including Implanon [26, 27]. However, studies conducted with the objective of exploring reasons of early removal of Implanon are quite limited. Therefore, this particular study was carried out in Arba Minch town to explore reasons for early removal of Implanon among users.

## Methods

### Study setting

The study was conducted in Arba Minch from April 20–27, 2018. Arba Minch is a town located in Gamo Gofa Zone of the Southern Nations, Nationalities, and Peoples Region about 500 km south of Addis Ababa. It is a town with an estimated total population of 74, 879, in which 39,208 are men and 35,671 are women. In the town, there are two health centers and one government hospital providing health services to the community. The town is administratively divided into 4 sub-cities and 11 kebeles (the smallest administrative unit).

### Study design

A community-based qualitative exploratory study using phenomenological approach was conducted. Phenomenological approach is a method of qualitative study in which the investigator identifies the human experiences concerning a phenomenon. It is a systematic and subjective approach to highlight and explain lived experiences and to further give them meaning. Understanding the lived experiences marks phenomenology as a philosophy as well as a method. In this process, the researcher works to take the experiences of participants on the participants’ own terms. This approach was selected as an important design to explore the reasons of early removal of Implanon among users [28].

### Study participants and sampling techniques

In this study, participants were selected from two different groups of population. The first group included women with at least one child and recently removed Implanon. Those women who removed Implanon in the past 12 months were considered as women who recently had Implanon removal. In this study, the discontinuers who used Implanon only for the duration of less than 2.5 years were eligible [23]. The second group included health extension workers (HEWs) who were closely working with those women in their respective villages. A total of 10 in-depth interviews with women who recently removed Implanon and 5 key informant interviews with HEWs were conducted. Among 11 kebeles found in Arba Minch town, 2 kebeles (Wuha Minch and Edget Ber kebeles) were selected purposively based on accessibility of clients with the experience of Implanon removal.

Five clients who had history of Implanon removal were selected purposively from each selected kebele for in-depth interviews. We used convenient sampling for selection of participants. This method helps to select few participants to learn more about their deep experiences. It allows capturing detailed explanations of all aspects of the phenomena under study. It also lets to capture important shared or latent patterns that may cut across cases in spite of their heterogeneity [29, 30]. Participants were invited for in-depth interviews at their convenient time and place. Five HEWs who were trained on Implanon insertion and were serving households in the above selected kebeles were included in the study purposively. The sample size was determined based on saturation point or the point at which no new information or themes are observed in the data or when the researcher is no longer hearing or seeing new information.

### Method of data collection and analysis

Data were collected through in-depth interview technique using an interview guide. The interview guide consists of questions on socio-demographic variables, client’s awareness on FP particularly about Implanon, reasons for early removal of of Implanon and post Implanon removal experience. The questions were not rigidly adhered to, but served as a guide for a structured conversation and to ensure all topics were covered. Before conducting the interview, ethical approval was obtained from Institutional Ethics Review Board (IRB) of College of Medicine and Health Sciences, Arba Minch University. Interviews were conducted in Amharic language. Free flow of information was encouraged through probing. The interview continued until information saturation is reached. The in-depth interviews were taped and note (key information) was taken by the investigator.

Audio data were transcribed verbatim into word files and translated from the Amharic language to English. The principal investigator reviewed key terms in Amharic language and their respective translations to ensure a degree of standardization. Final transcripts were compared against notes taken in order to ensure quality.

Before the analysis, the text was read through several times to be familiar with the data. The data were first saved in text format and imported into open code software version 4.0 to facilitate coding and categorizing. It was independently coded by two investigators (MMU and MK). The various codes were compared based on differences and similarities and sorted into categories. Finally based on content analysis, the underlying meaning that is the latent content of the text was formulated under each of the categories [31].

## Results

### Socio-demographic characteristics of study participants

A total of 10 in-depth interviews with Implanon users were conducted. All of the clients were married. About half of them belong to age group of 25–29. The mean age of the clients was 29.5 with a standard deviation of 5.3 years. The minimum and maximum ages of the participants were 20 and 35 years respectively. Except two, all of the participants were housewives by occupation. More than two thirds of the participants attended formal education. Only one of them had first degree. Seven of the participants had 3–5 births and almost all of the participants had no history of abortion. The participants had used Implanon for the duration of between 3 and 26 months with a mean of 9.2 ± 7.8 months. Five key informant interviews with health extension workers in the study villages were conducted. The in-depth and key-informant interview findings were combined, and presented in the following themes.

### Participants’ awareness on FP methods

This study showed that majority of participants were able to mention at least three types of FP methods available in nearby health facilities. These were Depo-Provera, pills, condom, implants and IUDs. Two participants were able to identify permanent contraceptive methods (male and female sterilization) used for birth limiting. Most of them were unable to mention the exact name of contraceptive methods they know. They tried to explain the methods in their own words.

*“… I know different types of contraceptives such as a method given for 3 months, contraceptives inserted in to hands / arms and loop. The methods are available in the nearby health facilities. I have the experience of using different types of contraceptives…”* (IDI_3_)

Another woman with the history of Implanon removal said that *“*…*I don’t know the names of contraceptive methods I ever used, but they are injectables (given for 3 months) and contraceptives inserted into the arm…”* (IDI_4_).

### Participants perceived benefits of Implanon

The study participants mentioned that implanon has a long term effect. Therefore, once it is inserted it doesn’t require our frequent remembrance and evades worry about unwanted pregnancy.

*“By using FP methods … I can prevent unwanted pregnancy … In using contraceptives inserted under the skin, there is no need of reminding daily or monthly. When we are using oral contraceptive pills and injectable there is a need to remember frequently.”* (IDI_6_)

This idea is also supported by one of the HEWs: *“They are using Implanon mainly because of its long term effect and believing that it has fewer side effects. They prefer it because it doesn’t require recalling every day.”* (HEW_1_).

### Counseling services related to Implanon insertion

Almost all clients received counseling services before Implanon insertion. Some of them indicated that the counseling was not adequate.

*“They (health professionals) have given me a counseling service before inserting Implanon. However, they didn’t inform me in detail about advantages and disadvantages of Implanon.”* (IDI_3_)

*“During the time of Implanon insertion, I didn’t get enough counseling from the health professional who provided me the service.”* (IDI_2_)

### Reasons for early removal of Implanon

Women who are using Implanon have the right to remove it at any time they want; however, unreasonably early removal is not advisable. This qualitative study identified various reasons for early removal of Implanon, in which side effect of the method was the main reason mentioned by participants. Particularly, heavy and irregular bleeding was the most frequently mentioned side effect. The others were headache, weight loss, skin disorders (itching, rash, dryness, and paleness), weakness (dizziness, difficulty of walking, physical weakness) and sick looking.

*“The main reasons for my early removal were heavy vaginal bleeding, weight loss, dizziness and difficulty of walking.”* (IDI_2_)

*“Even though my intention was to use Implanon for about 3 years, I decided to discontinue early because of the side effects. The side effects I experienced were heavy and irregular bleeding and weight loss.”* (IDI_9_)

Another participant indicated that husband opposition was one of the reasons for early removal of Implanon. “… *I had an intention to use Implanon for 3 years; however my husband was against my intention. He even quarreled with me and bitten me on my hands.*” (IDI_7_).

This idea was acknowledged by one of the HEWs. *“In my village there are some women who already have experienced early removal of Implanon. In my understanding the major reason is husband opposition. They say to their wives: you took it without my agreement; the side effect such as bleeding will cause a problem to you as it had caused to some other women and so that I can’t provide you extra food.”* (HEW_4_).

Seeking more children is also found to be the other reason for early removal of Implanon.

“… *even though I took it according to my personal choice and voluntary base, I have removed it after three months because of seeking more children.” (*IDI_6_).

“*The other reason for removal is a need for more children. Most of the time husband’s opposition comes either from the angle of side effects or from the need of more children.”* (HEW_1_)

The HEWs expressed method failure as one of the reasons for early removal of Implanon. Almost all HEWs explained that there were women who got pregnant while using Implanon. *“There were also mothers who removed Implanon due to unwanted pregnancy. I think it was either because of a problem related to drug potency or unnoticed pregnancy before insertion. Most of the time health professionals do not perform pregnancy test before inserting Implanon.”* (HEW_1_).

Furthermore, some of the HEWs articulated that some health professionals were forcing clients in making decision to use Implanon. Thus, this might also be a reason for early removal of Implanon. Whenever health professionals are decision makers in the use of Implanon, most users will end at early removal of it. “… *There is also other reason such as making a decision to use Implanon based on the choice of health professionals rather than clients. Most women who took Implanon by the choice of health professionals ended at early removal.”* (HEW_5_).

### The process of Implanon removal

This study revealed that majority of women requested for early removal of Implanon due to different reasons. However, some health professionals were hesitant and resistant to accept their request. Mostly they offered drugs and support for Implanon clients rather than accepting their request for removal. Generally, the process of removing Implanon in the public health facilities was very difficult.

“*In spite of my suffering from side effects, health professionals in public health facilities were resistant to remove.”* (IDI_1_)

*“Because of the side effects of Implanon, I was unable to continue until its expected time. Therefore, I decided to remove it early. Nevertheless, there was some kind resistant from the health professionals.”* (IDI_8_)

### Experience after Implanon removal

The study identified that most women regained their health immediately after removal of Implanon. Because of the side effects of Implanon, some of the study participants lost their desire to use any type of FP methods. Post removal of Implanon, most clients shifted to short acting contraceptive methods particularly to Depo-Provera.

*“After removal of Implanon, I regained my health and peaceful life”* (IDI_10_)

*“After removal of Implanon, I have not used any form of contraception. Depo-Provera is safe for most women, but is not for me. If there is any new, safe and effective contraceptive method, I will use it for the future otherwise I don’t want to use any of the available methods.”* (IDI_2_)

One of the HEWs stated that *“After removal of Implanon, most women are using injectable. They usually get it from the private clinics.”* (HEW_3_).

## Discussion

This study explored reasons for early removal of Implanon among users and found that awareness of participants about FP methods was almost universal, in which majority of participants were able to mention at least three types of FP methods that were available in nearby health facilities. The finding is far better than the findings of demographic and health surveys of Ethiopia that indicated women in reproductive age know at least one method of FP [7, 8].

In this study, heavy menstrual bleeding was identified as the main reason for early removal of Implanon. This finding is consistent with other prior studies conducted in Ethiopia, in which heavy menstrual bleeding or disturbance was reported to be the main reason for early Implanon removal [23–25, 27]. The finding of this study is also comparable with the findings of other previous studies conducted in South Africa and South Nigeria [32, 33].

Headache as side effect was also identified as a reason for early Implanon removal. It was verified in previous studies conducted in Ethiopia and South Africa [33, 34]. Contrary to evidences from other previous studies, weight loss was explored as one of the main reasons for Implanon removal [24, 34, 35]. This may be explained by individual and genetic differences.

In this and other studies conducted before, husband opposition was indicated as a reason for early Implanon removal [23–25, 27]. This may suggest gender inequality existing in developing countries including Ethiopia. This can also be observed in women’s decision making power in relation to their reproductive health [36–38]. Studies in other countries may substantiate husband’s opposition as one of the main reasons for early removal of Implanon [32–34].

In our study, seeking more children was identified as one of the common reasons for early removal of Implanon. This finding is consistent with other prior studies conducted in the country [23–25, 27, 35]. Previous Studies conducted in Nigeria and South Africa also confirmed that seeking more children resulted in high rate of discontinuation of Implanon [32–34]. This may be due to lack of proper pre-insertion counseling.

In the current study, there were some women with the experience of unwanted pregnancy while using implanon. This may be related to contraceptive failure [23, 34]. Furthermore, it can be associated with lack of pre-insertion counseling and screening for unnoticed pregnancy [33, 34].

This study explored some problems related to the process of Implanon removal. The process was more challenging due to poor link between health extension workers at community level and health professionals at health facility level. Accordingly, most clients encountered problems related to their reproductive health. This may be due to inaccessibility of removal services at the community level [14]. Furthermore, the process of Implanon removal has created uneasiness among clients, and enforced them to seek for removal outside of public health facilities [23, 27].

Post removal, some of the clients have regained their health, lost their desire of using any FP methods, or shifted to short acting FP methods, and / or experienced unplanned pregnancy. This finding is supported by finding from a study done in Butajira, Southern Central Ethiopia [25].

Our study was not without limitations. Though the sample is appropriate for the specific approach used in this study, the sample was small and limited to a single town. This restricted our ability to examine some aspects of our findings in detail. For instance, it was difficult to identify factors associated with experiencing method failure. The other limitation was the inability to involve health professionals from the nearby health facilities, as they were not part of this particular study.

## Conclusion

The findings of this study indicated that side effects of Implanon, husband opposition, seeking more children, and contraceptive failure were found to be reasons for early removal of Implanon, in which side effect of Implanon was the main reason. The findings of the study suggest the need for proper counseling service provision before Implanon insertion. Therefore, there should be adequate and all inclusive counseling program at all levels. There should also be strategies to increase male participation in FP activities in order that both partners will make decisions on their reproductive health together. Moreover, special consideration has to be given to access of services both for insertion and removal of Implanon based on their choice at any level. Furthermore, an advanced research should be conducted on large scale with mixed methods in order to better understand factors or reasons associated with early removal of Implanon.

## Data Availability

The datasets used and/or analyzed during the current study are available from the corresponding author on reasonable request.

## Abbreviations

EDHS: Ethiopian Demography and Health Survey
FP: Family Planning
HEWs: Health Extension Workers
IRB: Institutional Ethics Review Board
IUD: Intra-Uterine Device
MMR: Maternal Mortality Ratio
MOH: Ministry of Health
TFR: Total Fertility Rate
